# Identification and Validation of a Novel Immune-Related lncRNA Signature for Bladder Cancer

**DOI:** 10.3389/fonc.2021.704946

**Published:** 2021-07-12

**Authors:** Shan Hua, Zhiwen Xie, Wenhao Wang, Zhong Wan, Min Chen, Sheng Zhao, Juntao Jiang

**Affiliations:** ^1^ Department of Urology, Shanghai General Hospital, Shanghai Jiao Tong University School of Medicine, Shanghai, China; ^2^ Department of Urology, Shuguang Hospital, Shanghai University of Traditional Chinese Medicine, Shanghai, China

**Keywords:** urinary bladder neoplasms, long noncoding ribonucleic acids, signature, tumor-infiltrating immune cell, checkpoint blockade therapy

## Abstract

**Purpose:**

We aimed to construct an immune-related long noncoding ribonucleic acids (irlncRNA) signature to evaluate the prognosis of patients without specific expression level of these irlncRNA.

**Methods:**

The raw transcriptome data were downloaded from The Cancer Genome Atlas (TCGA), irlncRNAs were filtered out using an online immune related gene database and coexpression analysis, differently expressed irlncRNA (DEirlncRNA) pairs were identified by univariate analysis. The areas under curve (AUC) were compared and the Akaike information criterion (AIC) values of receiver operating curve (ROC) was counted, the most optimal model was constructed to divide bladder cancer patients into high- and low-risk groups usingõ the cut-off point of ROC. Then, we evaluated them from multiple perspectives, such as survival time, clinic-pathological characteristics, immune-related cells infiltrating, chemotherapeutics efficacy and immune checkpoint inhibitors.

**Results:**

14 DEirlncRNA pairs were included in this signature. Patients in high-risk groups demonstrated apparent shorter survival time, more aggressive clinic-pathological characteristics, different immune-related cells infiltrating status, lower chemotherapeutics efficacy.

**Conclusion:**

The irlncRNA signature demonstrated a promising prediction value for bladder cancer patients and was important in guiding clinical treatment.

## Introduction

Bladder cancer is a common malignant neoplasm with 81,190 new cases and 17,240 deaths having occurred in the USA in 2018, this categorization includes more than 700,000 living cases, and leads to approximately 150,000 deaths per year worldwide (1, 2). The main risk factor for bladder urothelial carcinoma (UC), which account for approximately 90% of all bladder cancers, is tobacco smoking; patients with a history of smoking have a 2.5 times elevated risk compared to non-smokers. Because of the fact that the impact of tobacco in the development of this disease possesses obvious hysteresis, regions with high incidence rates generally had a high smoking prevalence 20-30 years ago, including regions such as the US, Spain and other developed countries (3). Nonetheless, the immune checkpoint inhibitors (ICIs) can prevent the evasion of the immune system and the proliferation of cancer cells, and these inhibitors have revolutionized the treatment strategy of UC. The recent studies showed that the response to ICIs could be influenced by different immune cell infiltration. The infiltration of immune cells influenced the escape or evasion of the immune system for cancers to some extent (4).

Cisplatin has been approved by the Food and Drug Administration (FDA) for the systemic treatment of bladder cancer for over fifty years, and most clinical guidelines recommend that patients with muscle-invasive bladder cancer adopt neoadjuvant cisplatin-based chemotherapy (5, 6). Docetaxel has been proven to possess ideal antitumor activity for UC, regardless of whether it is used as a single agent or in combination with other chemotherapeutic agents. Additionally, it provides a salvage therapy when immunotherapy or checkpoint inhibitors are unsuitable for patients (7).

Long noncoding RNAs (lncRNAs), which refer to a type of RNA that is longer than 200 nt, account for approximately 80% of the human transcriptome (8, 9). They are mostly located in the nucleus and regulate gene expression *via* epigenetic regulation, transcriptional regulation and posttranscriptional regulation. Thus, some lncRNAs have the potential to act as biomarkers and therapeutic targets for many types of cancers, including UC (10). In recent years, lncRNAs have been proven to contribute to cancers through genomic or transcriptomic alterations, and they are able to affect the immune microenvironment because lncRNAs can cause tumor immune cell infiltration by regulating the expression of genes that are related to immune cell activation or cell lineage development (11, 12).

Recent studies have shown that immune infiltration signatures are likely to be promising tools to diagnose, evaluate, and treat a variety of cancers (13–16). LncRNAs play a significant role in the construction of these signatures. A recent study proved that an 11-lncRNA signature was a novel and significant prognostic factor for breast cancer (17). A 7-lncRNA signature associated with tumor immune infiltration has been identified *via* computational immune and lncRNA profiling analysis, and this signature was considered to be a predictive biomarker of ICI responses among non-small-cell lung cancer patients (15). Additionally, Zhang et al. established a 10 immune-related lncRNA signature that is associated with hepatocellular carcinoma (HCC) progression and prognosis for the prediction survival for HCC (18). A recent study provided a new immune gene-related lncRNA signature for distinguishing glioma groups, as well as for the diagnosis and treatment of glioma (19).

## Materials and Methods

### Retrieve Transcriptome Data and Identify Immune-Related Differentially Expressed lncRNAs

The raw transcriptome data of transitional cell papilloma and carcinoma types in bladder cancer were downloaded from TCGA (https://tcga-data.nci.nih.gov/tcga/). Afterwards, the raw data were annotated, and the lncRNAs were filtered out from the mRNAs by the human gene transfer format files that were obtained in Ensembl (http://asia.ensembl.org). The ImmPort online database offers a list of immune related rgenes, and the screening criteria coefficients of > 0.6 and a p-value of < 0.001 were set to identify irlncRNAs *via* coexpression analysis in R studio. Afterwards, the differentially expressed irlncRNAs between bladder cancer and normal paracarcinoma tissues were identified through the use of limma R package with the filter logFC > 1 and FDR <0.05.

### Construct DEirlncRNA Pairs

We compared each of the expression levels among the DEirlncRNAs that we have previously obtained, and constructed a 0-or-1 matrix with the criterion that the expression level of lncRNA A is higher than that of lncRNA B, which would provide a value of 1; otherwise, the value was 0. Then, the contrasted 0-or-1 matrix was further screened. We used survival R package with p < 0.01 as filter. And we could offer this code if necessary. If the lncRNA pairs containing any lncRNAs had no expression quantity (which indicates that these lncRNA pairs had no value for the prediction of survival outcomes), then these pairs were filtered out. The number of pairs with a value of 0 or 1 must have accounted for at least 20% of all of the irlncRNA pairs, unless they could not be used to construct the risk model.

### Retrieve Related Clinical Information of Patients

We downloaded clinical information of the bladder cancer patients in TCGA, and cases without complete clinical data, such as survival time, were removed.

### Calculate the Patients’ RiskScore With a Novel Risk Model

First, after the analysis of the single factor, a Lasso regression with 10 times cross proof was performed 1,000 times, and the p value was 0.05, in addition to 1,000 times of random stimulation for each time. We counted the number of occurrences in the Lasso regression for each irlncRNA pair; if the frequency of the irlncRNA pairs was greater than 100, then the pairs were further subjected to univariate and multivariate Cox hazard analyses and fourteen lncRNA pairs with p < 0.05 in multivariate Cox hazard analyses were used to construct the irlncRNA model. An area under curve (AUC) value for each model was calculated, and each curve was drawn. We obtained the maximum and ideal AUC values when the curve reached the peak, the programming statements were terminated, and the model was considered to be the most ideal at this time. We plotted 1-, 3- and 5-year receiver operating characteristic (ROC) curves and calculated their AUC values. The ROC curves reflecting the 1-, 3- and 5-year survival rates of bladder cancer patients were plotted, and their specific AUCs were calculated. The RiskScore was calculated with the use of the following formula: RiskScore = Σ^k^_i=1_βiSi. Patients with a RiskScore in the one-year ROC curve that was higher than the turning point (which was identified by calculating the Akaike information criterion [AIC] values) were regarded as having higher risks for dying in 5 years and were classified into the high-risk group; otherwise, the patients were classified into the low-risk group and were more likely to live longer than 5 years.

### Verify the irlncRNA Model in Clinical Conditions

A Kaplan-Meier analysis was used to compare the lifetimes of patients between the two groups, in order to validate of the cut-off point. We used R tools to visualize the survival curves and risk scores of every patient. For further clinical uses of this model, we used band diagrams to show the results of the chi-square tests, which explored the underlying connection between the clinicopathological characteristics and the model that we constructed. The risk score differences among the different clinicopathological characteristics were calculated *via* the Wilcoxon signed-rank test, and we showed the results graphically *via* box diagrams. Univariate and multivariate Cox regression analyses were performed to prove that the irlncRNA model was a valuable model to independently predict the prognosis of bladder cancer patients, and the results were illustrated *via* forest maps. R packages, including survival, ggplot2 and pHeatmap, were used to complete the previous analyses.

### Explore the Relevance Between Immune Cells and RiskScore

The immune infiltration status of all of the samples that were retrieved from the TCGA was calculated by using 7 methods, including XCELL (20, 21), TIMER (22, 23), QUANTISEQ (24, 25), CIBERSORT (26, 27), CIBERSORT-ABS (28), EPIC (29) and MCPcounter (30), to explore the relationship between immune-cell characteristics and risk. A series of Wilcoxon signed-rank tests were performed to compare the infiltrating immune cell content between the low- and high-risk groups, and box charts were used to show the results. We also explored whether risk scores of the patients were closely related to the infiltrated immune cells by calculating the Spearman rank coefficient, and we used lollipop diagrams to visualize the results. We set the threshold at p < 0.05.

### Identify the Relationship Between Chemosensitivity and the Risk Model

We used the half inhibitory concentration (IC50) of chemotherapeutic drugs, including gemcitabine, gefitinib, cisplatin and docetaxel, in the clinical cases from the TCGA as a reference value to assess the constructed model for clinical bladder cancer chemotherapeutic prognosis. Wilcoxon signed-rank tests were performed to investigate the difference in IC50 between the low- and high-risk groups, and the result is illustrated *via* box diagrams.

### Analyses of the Expressed Immunosuppressive Molecules Related to ICIs

In order to explore whether our risk model had significant relevance with ICI-related biomarkers express level, we visualize the results in violin plot using ggstatsplot R package.

## Results

### Retrieve Transcriptome Data and Identify Immune-Related Differentially Expressed lncRNAs

**Figure 1:**
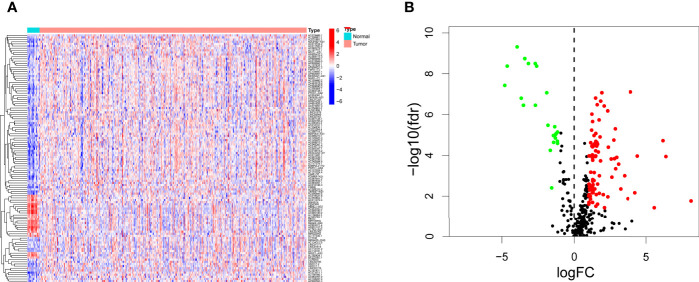
Differentially expressed immune-related lncRNAs (DEirlncRNAs) were identified using TCGA datasets and visualized in heatmap **(A)** and volcano plot **(B)**.

We downloaded transcriptome profiling data of bladder cancer from the TCGA, including 411 paracarcinoma normal samples and 19 tumor samples. We also downloaded GTF files in Ensembl to transfer Ensembl IDs to gene symbol IDs, and we performed a coexpression analysis between lncRNAs and irgenes (immune-related genes). In total, 315 irlncRNAs were identified, 116 of which were considered to be differentially expressed irlncRNAS (DEirlncRNA), 23 of which were downregulated and 93 of which were upregulated (**Table S1**, **Figures 1A, B**).

### Construct DEirlncRNA Pairs

**Figure 2:**
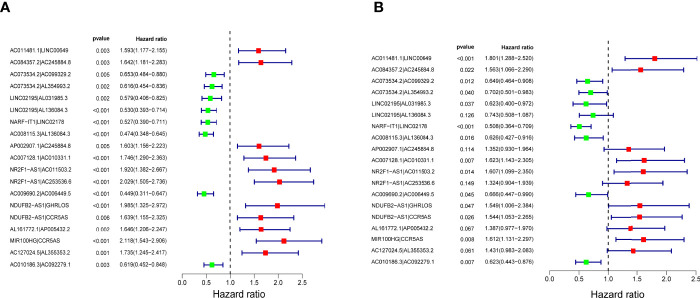
Nineteen lncRNA pairs were subjected to univariate **(A)** and multivariate **(B)** Cox hazard analyses. Fourteen lncRNA pairs with p < 0.01 were included in the model.

**Figure 3:**
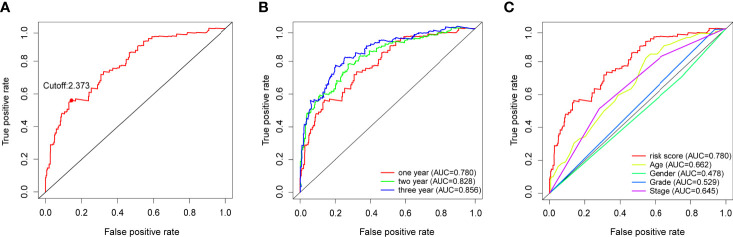
**(A)** Risk scores for 400 patients with bladder cancer; the maximum inflection point is the cut-off point obtained by the AIC. **(B)** The 1-,3-, and 5-year ROC of the model suggested that all AUC curves were over 0.780. **(C)** A comparison of 1-year ROC curves with other clinical characteristics showed the superiority of the risk scores.

The 116 DEirlncRNAs were identified with the limma R package in R studio, and 4,561 pairs were identified. A total of 467 pairs were regarded as being valid, and a univariate Cox model was performed. Fourteen pairs (p < 0.05) were used to construct a multivariate Cox model using stepwise regression (**Figures 2A, B**). Afterwards, we constructed the ROC curve of 14 pairs of irlncRNAs, and the AUC of the ROC curve that was 0.780, which was used to identify the most valuable lncRNA pairs to construct the most satisfying risk model. We calculated that the maximum cut-off point (the maximum inflection point) on the 5-year ROC curve, was 2.373 (**Figure 3A**). In addition, the AUC values for the one-, three- and five-year ROC curves were 0.780, 0.828 and 0.856, respectively (**Figure 3B**). Additionally, ROC curves including 5 years and other clinical characteristics were drawn to compare the optimality of our model, and the AUC value of the risk score was much higher than that of the others (**Figure 3C**). The clinical data of 409 bladder cancer cases were retrieved from the TCGA, and 400 of cases were valuable after removing the samples without incomplete data. The cut-off point (2.373) of the risk score divided all of the samples into two groups: high-risk and low-risk groups.

### Verify the irlncRNA Model in the Clinic

**Figure 4:**
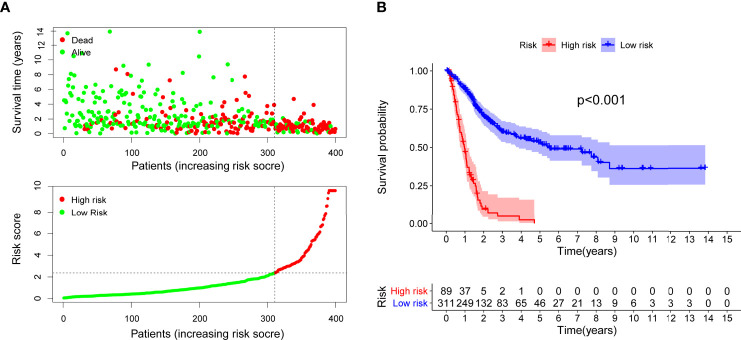
Risk scores **(A)** and survival outcome **(B)** of each case are shown. Patients in high-risk group had a shorter survival time tested by the Kaplan-Meier test.

**Figure 5:**
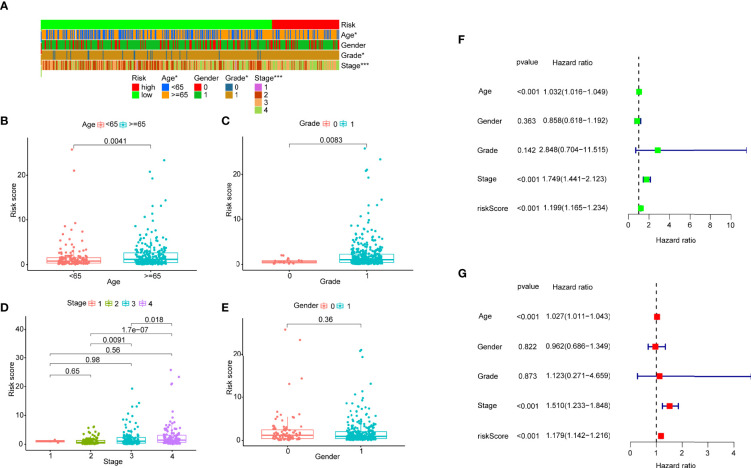
**(A)** A strip chart and the scatter diagram demonstrated that **(B)** age, **(C)** tumor grade, **(D)** T stage and **(E)** gender were apparently associated with the riskScore. 0 represent female, 1 represents male. **(F)** A univariate Cox hazard analyses showed that age (p < 0.001, HR = 1.032, 95% CI [1.016–1.049]), T stage (p < 0.001, HR = 1.749, 95% CI [1.441-2.123]), riskScore (p < 0.001, HR = 1.199, 95% CI [1.165-1.234]) were statistically different, and age (p < 0.001, HR = 1.027, 95% CI [1.011–1.043]), T stage (p < 0.001, HR = 1.510, 95% CI [1.233-1.848]), riskScore (p < 0.001, HR = 1.179, 95% CI [1.142-1.216]) were statistically different in multivariate Cox hazard analyses **(G)**.

After distinguishing between the groups, 89 cases were considered to be high-risk, and 311 cases were considered to be low-risk. Two diagrams illustrated the risk scores and survival times for all of the samples (**Figure 4A**). More patients died, and the survival time apparently decreased as the risk score increased. The Kaplan-Meier analysis and the corresponding survival curves showed that the patients with low riskScores lived much longer than patients with high riskScores (p < 0.001) (**Figure 4B**). Additionally, the analysis and curved showed that almost all of the patients with high RiskScores lived fewer than 5 years, and that approximately 50% of the patients in the low-risk group remained alive then. In order to explore the relationship between the clinicopathological characteristics and the risk of bladder cancer, we performed a set of chi-square tests, including age, sex, grade, stage and risk scores. The strip charts show the overall results, and age, tumor grade and tumor stage exhibit extremely close relationships with risk (**Figure 5A**). Additionally, age (**Figure 5B**), tumor grade (**Figure 5C**) and tumor stage (except stage I) (**Figure 5D**) exhibit extremely close relationships with risk, whereas males and females had the same risk (**Figure 5E**). Additionally, we proved that age (p < 0.001, HR = 1.032, 95% CI [1.016-1.049]), tumor stage (p < 0.001, HR = 1.749, 95% CI [1.441-2.123]) and RiskScore (p < 0.001, HR = 1.199, 95% CI [1.165-1.234]) exhibited significant statistical differences in the univariate Cox regression analysis, and age (p < 0.001, HR = 1.027, 95% CI [1.011-1.043]) (**Figure 5F**), tumor stage (p < 0.001, HR = 1.510, 95% CI [1.233-1.848]) and RiskScore (p < 0.001. HR = 1.179, 95% CI [1.142-1.216]) were also significantly different in the multivariate Cox regression analysis (**Figure 5G**).

### Estimating Tumor-Infiltrating Immune Cells and Immunosuppressive Molecules With a Risk Assessment Model

**Figure 6:**
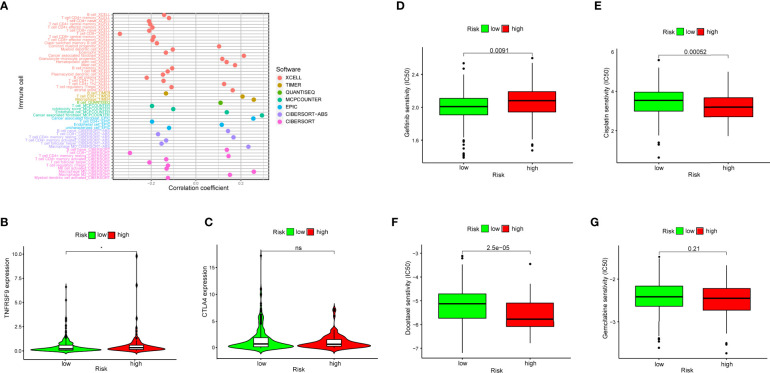
**(A)** Spearman correlation analysis showed patients in the high-risk group were more positively associated with tumor-infiltrating immune cells, such as fibroblasts, endothelial cells, monocytes, macrophages and neutrophils. **(B, C)** High risk scores were positively associated with upregulated TNFRSF9 expression level, whereas CTLA4 level had no difference in high- and low-risk groups. **(D–G)** The model could be a predictor for chemosensitivity as higher risk scores had different IC_50_ for chemotherapeutics such as gefitinib, cisplatin, docetaxel and gemcitabine.

Due to the fact that the irlncRNA model was initially related to immune-related genes, we then explored the relevance of this model to the immune microenvironment. A series of Wilcoxon signed-rank tests indicated that more immune cells, including fibroblasts, endothelial cells, monocytes, macrophages and neutrophils, infiltrated the tumor microenvironment in patients with a higher RiskScore, whereas the low-risk group was negatively associated with activated myeloid dendritic cells, CD4+ T cells and eosinophils (**Figure S1**). Some types of cells with samples sizes that were too small or that had opposite results in different databases were removed. The diagram illustrating the result of the Spearman correlation analysis is summarized in **Figure 6A**. Additionally, we explored whether our model had relevance to ICI-related gene expression and found that patients in the high-risk group had a positive correlation with a high expression level of TNFRSF9 (p < 0.05) (**Figure 6B**), whereas CTLA4 did not exhibit a significant difference (**Figure 6C**). One major reason for this may be due to the small sample size.

### Identify the Relationship Between Chemosensitivity and the Risk Model

To investigate whether the efficacy of some frequently-used chemotherapeutics was associated with risk, we compared the common drug sensitivity (which was represented by IC50) between patients with low- or high- risk scores. The results showed that high-risk patients had a higher IC50 for gefitinib (p = 0.0091) (**Figure 6D**), and a lower IC50 for cisplatin (p = 0.00052) (**Figure 6E**) and for docetaxel (p < 0.0001) (**Figure 6F**), whereas there was no significant difference for gemcitabine (p = 0.21) (**Figure 6G**). Therefore, this suggested that the irlncRNA model could be used as a predictor for chemosensitivity.

## Discussion

The most of recent studies have focused on the establishment of signatures for RNAs (other than protein-coding RNAs) to predict the prognosis of patients with malignancies (31). Motivated by the significance of irlncRNAs, we attempted to establish an applicable signature with DEirlncRNA combinations to investigate potential functions regarding immunotherapy response in this study.

First, we identified immune-related genes by processing raw data that were retrieved in the TCGA and subsequently further performed a coexpression analysis, as well as a differentially expressed analysis to classify the irlncRNAs. Second, followed by screening with an iteration loop and a 0-or-1 matrix, a univariate Cox regression analysis (combined with a multivariate Cox regression analysis) was used to determine the DEirlncRNAs signature. We also used an independent prognostic analysis to incorporate the risk scores and other clinical parameters for the validation of whether these indicators were capable of independently distinguishing the outcome. After discriminating the high- and low-risk groups by the cut-off value of the risk scores, we calculated the AUC value of the ROC curve at 1-, 3- and 5-years to validate the candidate signature, as well as evaluating the survival outcome. Consequently, we investigated the correlation between the difference in the RiskScore under this novel signature and several common clinicopathologic features. Variances in intratumoral immune infiltrating cells have a profound impact on the treatment responses to immune checkpoint inhibitors (32). To investigate the relationship between the RiskScore and immune infiltrating cells, we used several commonly known approaches to calculate the status of the immune infiltrating cells. Based on the immune microenvironment analysis, it is reasonable to personally imply therapeutic benefits from chemotherapy and immunotherapy of each patient through the use of immune scores (33). In addition, previous studies have indicated that the modulating of the immune microenvironment may be essential for improving the radiotherapy-induced antitumor response (34). Luo et al. reported that tumor mutation burden (TMB) was associated with the infiltration of activated CD4(+) memory T cells in the immune microenvironment (35). Our signature also indicated that, a high- risk score was negatively associated with a high sensitivity to chemotherapeutic drugs, such as cisplatin, gemcitabine, and docetaxel, rather than to the commonly administered chemotherapeutics in bladder cancer, such as gefitinib. Though the signature was only significantly associated with ICI-related biomarkers like TNFRSF9, instead of CTLA4, LAG3, HAVCR2 and PDCD1, thus proving that the efficacy of immunotherapy still presents an underprivileged position within the treatment of bladder cancer. However, we believed that the specific mechanism and biomarkers should be identified and validated, due to the different subtypes of immune infiltrating cells and the immune-related functional phenotypes in bladder cancer.

Our algorithm helped us identify DEirlncRNAs and construct the most significant irlncRNA pair. We could detect the pairs with higher or lower expression rather than examining the exact expression levels of every irlncRNA. In addition, lncRNAs we identified were associated with immune genes, which remodel the immune microenvironment and active immune cells. Thus, our signature had an advantage of clinical practicability to distinguish high or low risk for bladder cancer patients. To get a more accurate prediction on risk for patients, a modified method called Lasso penalized modeling was performed. Besides, every AUC value used to identify the best model followed by comparison with other common-used clinical parameters, the AIC values were used to get the best cut-off point for model fitting rather than distinguishing just by the median value. We also revaluated the survival outcome, analyzed the efficacy of some chemotherapeutics, tumor infiltration and ICIs to prove that our signature worked well.

However, there were some limitations of our study. Specifically, one example was that the data in the TCGA project were relatively insufficient for identifying initial irlncRNAs, and datasets from another independent database were required. Therefore, the constructed signature requires validation from an external database because the expression levels vary in each case. Unfortunately, we failed to implement the validation of the entire signature and the survival outcomes because of the failure to retrieve ideal GEO datasets. Consequently, we plan to collect clinical samples for RNA-seq, verify our risk model in future experiments, as well as to establish more reliable clinical connections for this novel signature. Moreover, we can divide these patients with the combination of the RiskScore and other characteristics to achieve more accurate and personalized prognosis judgements.

A variety of methods were performed to confirm this novel algorithm, which was used to establish our signature in this study, so we believed our signature was of significance in spite of the lack of further validation. However, external validation could be beneficial.

## Conclusion

LncRNAs have been proven to possess good prognostic value and can be potential therapeutic targets for many malignant tumors. In this study, we constructed a novel 14-irlncRNA-pairs signature to evaluate the prognose of bladder cancer patients. Patients were able to be divided into high- and low-risk patients *via* our signature, and patients in the high-risk group had a much shorter survival time. This signature showed obviously better prognostic value than other clinicopathological characteristics, such as age, tumor stage and tumor grade. Additionally, the patients’ sensitivities to chemotherapeutic drugs and the infiltration of immune cells in the tumor microenvironment showed significant differences between the patients in the high- and low-risk groups. In summary, our novel signature may be a valuable predictor for the prognosis of bladder cancer patients and can be used for chemotherapy drug selection the clinical settings in the future. However, the verification in further experiments or other datasets can also be meaningful for this irlncRNA signature.

## Data Availability Statement

The original contributions presented in the study are included in the article/**Supplementary Material**. Further inquiries can be directed to the corresponding author.

## Author Contributions

JJ conceived and funded the study. SH designed and performed the research, drafted the manuscript. WW and ZX performed and drafted the manuscript. MC and SZ coordinated technical support. ZW revised the manuscript. All authors contributed to the article and approved the submitted version.

## Funding

This work was supported by The National Natural Science Foundation of China (No.81771564).

## Conflict of Interest

The authors declare that the research was conducted in the absence of any commercial or financial relationships that could be construed as a potential conflict of interest.
