# Supplementary figures and images for: Identification and Validation of a Novel Immune-Related lncRNA Signature for Bladder Cancer
Source: Front Oncol. 2021 Jul 12;11:704946. doi: 10.3389/fonc.2021.704946 (PMC8311739; doi:10.3389/fonc.2021.704946)

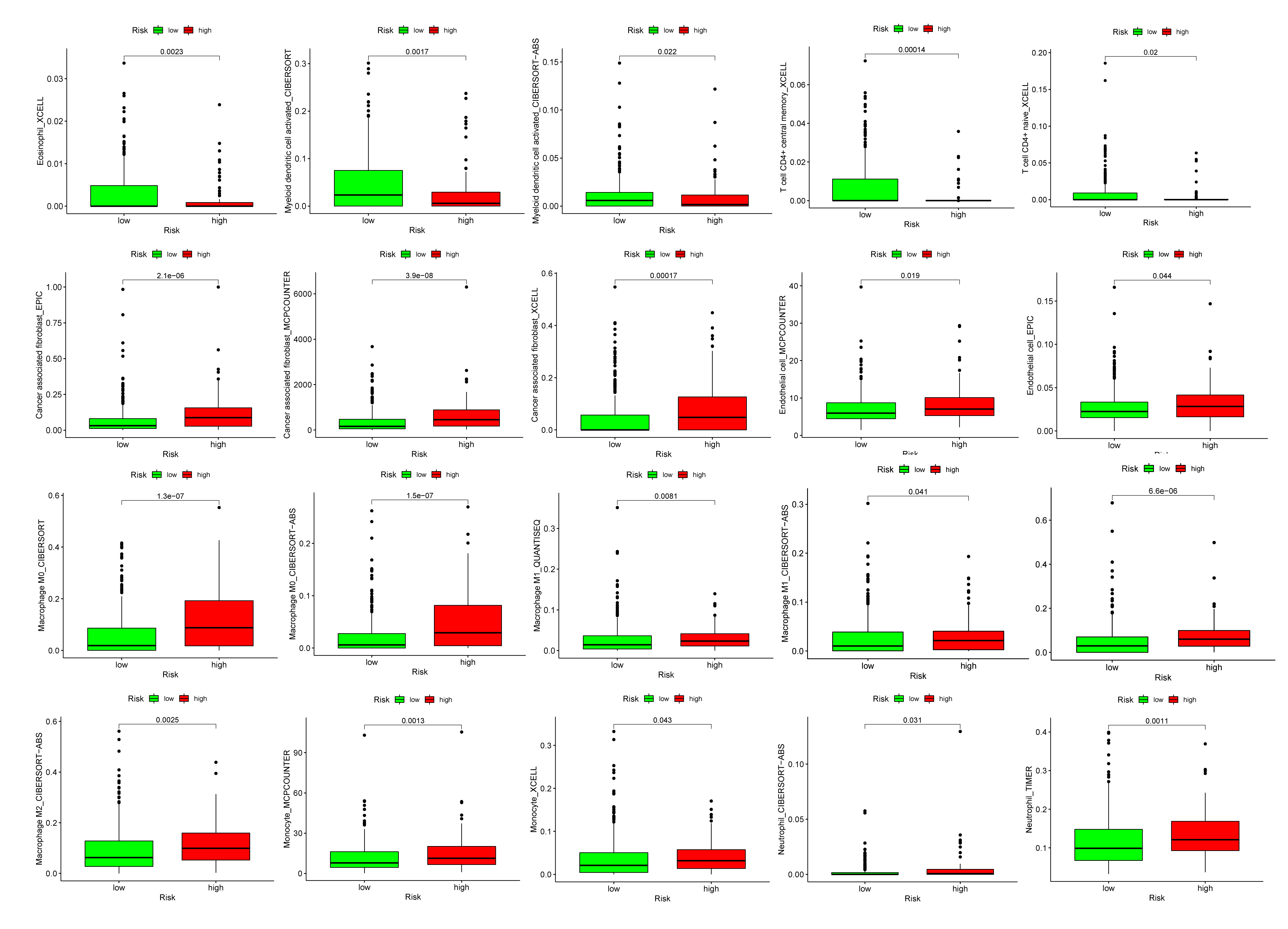

Supplement: Supplementary file 1 [file Image_1.tif]
